# Perspectives on heterogeneity-informed cognitive training for attention-deficit/hyperactivity disorder

**DOI:** 10.3389/fpsyt.2022.1100008

**Published:** 2023-01-12

**Authors:** Da-Wei Zhang

**Affiliations:** ^1^Department of Psychology and Center for Place-Based Education, Yangzhou University, Yangzhou, China; ^2^Department of Psychology, Monash University Malaysia, Bandar Sunway, Malaysia

**Keywords:** ADHD, cognitive training, heterogeneity, training protocols, optimization

## Abstract

Attention-deficit/hyperactivity disorder (AD/HD) is a heterogeneous neurodevelopmental condition, posing a severe threat to quality of life. Pharmacological therapies are the front-line treatment; however, their shortages encourage the development of alternative treatments for AD/HD. One promising method of developing alternative treatments is cognitive training (CT). A CT-based therapy was recently approved by the US Food and Drug Administration. However, due to heterogeneity in AD/HD, a CT protocol is unlikely to provide a one-size-fits-all solution for all patients with AD/HD. Therefore, this article highlights key aspects that need to be considered to further develop CT protocols for AD/HD, regarding training content, timing, suitability, and delivery mode. The perspectives presented here contribute to optimizing CT as an alternative option for treating AD/HD.

## 1. Introduction

Attention-deficit/hyperactivity disorder (AD/HD), a common neurodevelopmental disorder (1), poses a severe threat to quality of life (2). AD/HD is heterogeneous in etiology, with both hereditability and environmental factors contributing to the condition (3). Pharmacological therapies are the front-line treatment (4, 5) but have been criticized for their side effects (5, 6), lack of long-term effect (7), and economic impact (8). Thus, developing alternative treatments for AD/HD is of great importance.

Recently, cognitive training (CT) has become an alternative option for treating AD/HD. CT, a concept analogous to physical training, is a non-pharmacological approach and provides an array of opportunities for developing desired cognitive skills (9). It has been shown that cognitive impairment can contribute to AD/HD symptoms (10, 11), but that it can also be malleable (12, 13). Based on these, CT aims to alleviate AD/HD symptoms by improving impaired cognitive abilities. Two decades have passed since CT was introduced in AD/HD (14). Kollins et al. (15) reported a landmark study in this field, which demonstrated that a 4-week CT protocol, known as AKL-T01, significantly improved inattention in children with AD/HD with a multi-center randomized control trial. This promising result led to AKL-T01 being approved by the US Food and Drug Administration (FDA) for treating inattention in children with AD/HD—the first FDA-approved digital therapeutics in AD/HD (16).

While CT has become a treatment option, heterogeneity in AD/HD can affect the effect of CT and should be considered for further developing CT protocols. AD/HD is well-known for its heterogeneity in etiology and phenotype (3). Even for the same patient, AD/HD symptoms can fluctuate and result in secondary mental disorders over time (3, 17). Due to heterogeneity, patients with AD/HD respond differently to a range of treatment options, for example, pharmacological therapies (18), neurofeedback training (19), and trigeminal nerve stimulation (20). Similarly, a recent study reported that children with AD/HD differed in their response to CT (21). Together, due to the complexity caused by heterogeneity, a CT protocol is unlikely a one-size-fits-all solution for AD/HD. However, the role of heterogeneity has not been adequately considered. Therefore, this article highlights key aspects that need further consideration to optimize CT as a treatment for AD/HD, regarding training content, timing, suitability, and delivery mode.

## 2. Beyond executive function training

The multifaceted nature of cognitive deficits in AD/HD requires CT to target a variety of cognitive abilities. Multiple cognitive deficits have been reported in AD/HD at the group level, including sustained attention, executive functions (EF), and motivational functions (10, 11). These cognitive functions are mediated by unique brain mechanisms (11, 22) and lead to different aspects of AD/HD symptoms (11). CT should therefore be directed at addressing these cognitive deficits.

However, few CT protocols considered motivational deficits in AD/HD. Motivation can be incorporated in CT for AD/HD in at least two ways. Firstly, CT protocols can be tailored to train impaired motivational processing in AD/HD. The preference for immediate small rewards over delayed large rewards—the delay gratification issue—is a well-recognized motivational deficit in AD/HD (11). However, a glance at review papers reveals that previous CT protocols for AD/HD mainly targeted sustained attention and executive function (EF) but rarely trained this motivational deficit [e.g., (23)]. In typically developed children, there have been successful attempts to delay gratification by reducing undesired thinking (24). Future studies may explore whether this CT protocol designed to delay gratification can benefit those with AD/HD. Secondly, CT protocols can leverage motivation to boost training of sustained attention and EF. The effectiveness of CT requires multiple sessions of intensive training, which can be challenging for AD/HD as evidenced by the high dropout rate in previous studies [e.g., (25, 26)]. Boosting motivation is an immediate solution to this problem. Motivational brain networks interact with the brain networks underlying sustained attention and EF such that motivation can prompt more efforts to sharpen sustained attention and EF (22). Consequently, CT protocols may involve features to boost motivation for facilitating or enhancing training effects. It has been demonstrated that CT protocols incorporating motivational elements (e.g., gamification) produced better training effects in healthy populations (27, 28). Some CT protocols for AD/HD indeed incorporated motivational features, such as gamifying training tasks and providing immediate feedback (29, 30). However, it should be noted that motivational features (e.g., changing game themes and daily prizes) may become distractors instead and negatively impact training improvement in typical children (31). Since AD/HD is more susceptible to distractors, inappropriate motivational features may have a more significant detrimental impact. Together, while incorporating motivational design into CT is an effective way to facilitate training effects in AD/HD, future research should examine how to optimize motivational features.

Beyond rehabilitation (e.g., training on motivation and EF), cognitive training may contribute to academic development in AD/HD by improving domain-specific academic skills. Schooling can be particularly challenging for AD/HD. Although AD/HD is not a learning disability, those with AD/HD typically experience learning difficulties beginning in their early schooling years (32). Early learning difficulties adversely affect the development of fundamental domain-specific academic skills, such as mathematical facts retrieval (33) and spelling (34), which in turn negatively impacts their long-term academic achievement (35). In typical children, there has been evidence that specific forms of CT can improve domain-specific academic skills. For example, fundamental mathematical and reading skills can be improved by “number line training” (36) and “phonemic awareness training” (37), respectively. Thus, future studies may examine whether cognitive training targeting domain-specific academic skills is superior to the “teaching as usual” condition in improving early academic skills and easing early learning difficulties in children with AD/HD.

Regardless of training purposes, impurity in training content should be considered. Psychometric studies have shown that cognitive task paradigms, particularly those used for EF, involve not only the targeted cognitive ability but also incidental cognitive abilities (38). The color-word Stroop task, for example, is expected to tax inhibition but also involves semantic and sensory processing. The issue is known as task impurity (38). CT is typically developed based on the task paradigms for measuring desired cognitive abilities. Due to task impurity, repetitive CT also increases exposure to non-target cognitive abilities. An unwanted consequence is that trainees primarily focus and improve on non-target cognitive abilities. One possible solution to the issue is to develop CT protocols based on multiple task paradigms that heavily tax the desired abilities. This is based on the rationale that the impurity component is task-specific (non-overlapping between tasks), whereas the desired component is task-general (overlapping between tasks) (38). Hence, CT based on multiple task paradigms minimizes the involvement of non-target abilities across sessions.

## 3. Toward personalized CT

The cognitive deficits exhibited at the group level (e.g., ADHD vs. controls) do not necessarily indicate that all patients with AD/HD suffer the same deficits. Instead, cognitive deficits vary among those with AD/HD (39). Some patients with AD/HD may have impaired sustained attention, while others may have typical sustained attention but a deficient subcomponent of EF.

The heterogeneous cognitive profiles offer insight into who may benefit most from CT in AD/HD. In a working memory training program, children with AD/HD who had poorer working memory before training (baseline) improved more (21). The result is in line with the pattern displayed from other types of cognitive interventions in children—a poorer baseline predicts greater training improvement (40). These findings suggest that CT in AD/HD should be tailored to the deficient type, which is particularly important since there may be various CT protocols (e.g., attention-focused CT vs. motivation-focused CT).

Although the baseline-improvement pattern seems intuitive, it is in contrast to what has been found in typically developed populations—a poorer baseline predicts smaller improvement in CT (41, 42). The discrepancy has not been well-explored. One possible explanation is that CT acts on different mechanisms in different populations. A capacity-efficiency model has been developed to explain CT mechanisms (9). For people who have not fully developed their cognitive capacity (e.g., AD/HD), training might act on the capacity mechanism where CT enhances the capacity of overall cognitive resources (43). Poor baselines, in this case, indicate more room for improvement. Thus, the poorer-greater pattern is observed. Conversely, CT may act mainly on optimizing cognitive performance within the existing capacity limit for people with fully developed cognitive abilities (e.g., typical adults). Poor baselines indicate a lack of knowledge and sources to optimize cognitive performance, resulting in the poorer-poorer pattern.

Neuroimaging profiles may provide unique value in identifying who might benefit from CT in AD/HD. After controlling baseline cognitive profiles, baseline brain structural profiles were found to predict additional CT improvement in typically developing children (41). This may also apply to those with AD/HD. Additionally, neuroimaging profiles have been shown to uniquely predict improvements in other interventions (20). Future studies may examine whether combining cognitive profiles with neuroimaging profiles helps identify those with AD/HD who are most likely to benefit from CT.

The question of who might benefit from CT prompts responder analysis but deserves methodological consideration. Selecting the statistical model is one of the issues. Responder analysis typically begins with defining responders and non-responders based on the difference between the pre- post-intervention change. The cut-off point is arbitrary (e.g., the 30% as responders and the bottom 30% as non-responders). Then, the defined responders are characterized by a group comparison (responder vs. non-responders) or regression analysis. However, this method of determining responders may suffer regression toward the mean because of measurement errors [for more discussion and alternative models, please see Castro-Schilo and Grimm (44) and (30, 45)]. The second issue refers to generalizability. Characterizing responders is a prediction problem. Most studies have addressed this issue by using in-sample regression analysis, which primarily reflects correlations between variables within a sample and does not necessarily generalize to other samples (46). There have been recommendations (e.g., k-fold cross-validation and large sample sizes) for ensuring the validity of predictive models (46).

## 4. Age matters

Childhood is a unique window for conducting CT in AD/HD. Childhood is a sensitive period for developing cognitive skills because of its greater neuroplasticity (47, 48). CT has been shown to lead to larger cognitive development in children (49). In the same vein, the advantage of childhood may also enhance the effectiveness of CT as a treatment for reducing AD/HD symptoms.

The developmental course of cognitive deficits in AD/HD also favors CT in childhood. Developmental cascades refer to the effect that the development of a cognitive function at one time point consequently affects the emergence or growth of other cognitive functions at later time points (50). The effect of developmental cascades can be adverse. In AD/HD, for instance, atypical cognitive development at an early age may eventually lead to various cognitive deficits or secondary mental disorders at an older age (3, 51). Following this developmental perspective, CT should be delivered as early as possible to avoid the adverse effect of developmental cascades.

The question then arises as to how early CT can be performed. A recent study examined the feasibility of CT intervention for infants aged 9–16 months (52). After easing the concern on feasibility in young children, the question is therefore reduced to how early AD/HD can be reliably diagnosed. Although AD/HD is usually diagnosed at school age, genetic progress may enable an earlier diagnosis. Using the genome-wide association study, researchers have identified several genetic loci associated with AD/HD symptoms [e.g., (53)]. The genetic pattern implies the possibility of conducting preemptive CT for individuals genetically predisposed to AD/HD to prevent the detrimental cascades. However, preemptive interventions in AD/HD will also be subject to the ethical issues related to preemptive interventions in other neurodevelopmental disorders, for example, neglecting the effect of “nurture” and miscommunicating early concerns to families [for further discussion, please see Manzini et al. (54)].

The advantage of early CT does not preclude the possibility of CT for adults with AD/HD. As neuroplasticity is a life-long brain property, adults with AD/HD are also expected to benefit from CT. Despite this, few studies have examined the efficacy of CT in adults with AD/HD, and most of them reported negative results (55–59). There are two possible reasons for the discouraging results. The existing studies primarily used CT protocols that were developed for children. This may not be appropriate, given that the dropout rate for adults is greater than that for children (25). The high dropout rate also resulted in insufficient statistical power to detect training effects (58). The second possible reason is that previous CT studies mainly targeted working memory (55–58), while inattention is the dominant symptom in adults with AD/HD (60, 61). Therefore, CT protocols focusing on attention may be more effective in treating adults with AD/HD. Promising outcomes were indeed reported for attention-based CT in adults with AD/HD (59).

## 5. Not only computerized CT

In terms of delivery, CT can be broadly divided into computerized CT and non-computerized CT (23). Computerized CT delivers training content using digital devices (e.g., computers and smartphones). Training content is typically gamified to increase training adherence, for example, using a gamified Go/Nogo task to train inhibition (29, 30). Non-computerized CT is a broader term, encompassing multiple forms, such as board games based on the Corsi block task to train working memory (23) and physical activity based on the “Simon Says” task to train inhibition (62). Computerized CT offers the advantages of precisely controlling training content (e.g., training duration and difficulty) and requires less manual guidance, whereas non-computerized CT is more cost-effective and ecological (45).

Due to the difference in delivery methods, CT may target different aspects of the same cognitive construct. Many CT protocols target EF, as described above. Previous studies have reported that EF measured by computerized cognitive tasks is not or is weakly correlated with those measured by questionnaires [e.g., (63)]. In light of these findings, a distinction is made between “cold” and “hot” EF (e.g., those measured by computerized tasks or questionnaires) (64). The former tax more on “pure” EF, whereas the latter reflects the capacity of EF in everyday life where other cognitive processes (e.g., emotion and motivation) are also involved (64). Non-computerized CT has been suggested advantage in improving “hot” EF (65). It is possible that computerized and non-computerized CT protocols target EF from different aspects—“cold” EF or “hot” EF. However, few studies have examined this issue to date. Future studies should systematically compare the two types of CT in terms of their effects on “cold” and “hot” EF. The comparison could optimize the delivery mode of CT in AD/HD since some patients with AD/HD have impaired “cold” EF, while others have impaired “hot” EF (66).

Combining CT with other interventions is another promising delivery mode. One reason for this combination is to improve the transferability of training effects in specific settings. Compared to CT alone, CT combined with supported employment programs has been shown to generate larger training effects in vocational settings in other populations (67). This combined protocol may also be beneficial to AD/HD with poor vocational skills. The transfer effect of CT is often explained by the common element theory (68, 69). In this regard, the combined protocol is superior because it discloses the similarity between skills learned in CT and skills required in specific settings. Secondly, CT can be used in conjunction with interventions that enhance neuroplasticity. This combination is grounded on the points that neuroplasticity is a prerequisite for CT improvement, and interventions that enhance neuroplasticity can thus improve training effects (45). Following this perspective, attempts have been made to combine CT with non-invasive brain stimulation; however, the results are inconsistent [see Dakwar-Kawar et al. (70) but also Westwood et al. (71)]. The disparity may be due to the different timing and intensity at which brain stimulation was delivered, as the impact of the same brain stimulation on neuroplasticity varies with both time and intensity (45, 72). Further exploration of the mechanisms underlying non-invasive brain stimulation may lead to more effective combinations.

## 6. Conclusion

Medicines are usually accompanied by a lengthy guide that describes the factors affecting their effectiveness. While FDA has approved one CT protocol as a treatment for AD/HD, the key message from this article is that heterogeneity in AD/HD affects what, who, when, and how CT should be performed. The complexity of AD/HD suggests that one CT protocol is unlikely to provide a one-size-fits-all solution for AD/HD, thus appealing to “heterogeneous” CT protocols.

## Data availability statement

The original contributions presented in the study are included in the article/supplementary material; further inquiries can be directed to the corresponding author.

## Author contributions

D-WZ participated in the conceptualization, writing, and editing process of this manuscript.
